# Targeting MGLL: Terazosin Regulates Triglyceride Metabolism to Mitigate Endothelial Cell Senescence

**DOI:** 10.1093/geroni/igaf122.2894

**Published:** 2025-12-31

**Authors:** Jie Huang, Jinghua Yan, Hao Nie, Cuntai Zhang

**Affiliations:** Tongji Hospital, Tongji Medical College, Huazhong University of Science and Technology, Wuhan, Hebei, China (People’s Republic); Tongji Hospital, Tongji Medical College, Huazhong University of Science and Technology, Wuhan, Hubei, China (People’s Republic); Tongji Hospital, Tongji Medical College, Huazhong University of Science and Technology, Wuhan, Hubei, China (People’s Republic); Huazhong University of Science and Technology, Wuhan, Hubei, China (People’s Republic)

## Abstract

Metabolic disorders often arise in senescent endothelial cells, impairing endothelial function, leading to diminished vasodilation and increased vascular stiffness, contributing to the development of cardiovascular disease (CVD). Despite notable advancements, the molecular mechanisms driving endothelial cell senescence and its contribution to vascular aging remain poorly understood. This knowledge gap hinders the development of effective therapeutic strategies. This study investigates the role of terazosin (TZ) in mitigating vascular endothelial cell senescence, with emphasis on its impact on glycerolipid metabolism. Using both in vivo (aged mice) and in vitro (human umbilical vein endothelial cells, HUVECs) models, the research employs techniques such as senescence-associated β-galactosidase (SA-β-gal) staining, lipidomics analysis, and molecular docking simulations to uncover the underlying mechanisms. The results demonstrated that TZ treatment could significantly improve endothelial-dependent vasodilation, reduce vascular stiffness, and attenuate senescence markers in aged mice. Lipidomics analysis revealed that TZ could modulate glycerolipid metabolism, specifically by reducing palmitic acid accumulation in senescent endothelial cells. Further investigation identified monoglyceride lipase (MGLL), which hydrolyzed monoglycerides into palmitic acid and glycerol, as a key player in endothelial cell senescence. TZ reduced palmitic acid accumulation by targeting MGLL, thereby mitigating endothelial senescence. In conclusion, this study provides novel insights into the anti-senescence effects of TZ, highlighting its potential as a therapeutic agent for age-related vascular diseases. Moreover, the identification of MGLL as a critical regulator of glycerolipid metabolism and endothelial cell senescence offers a promising target for future interventions in vascular aging.

